# Predicting the Risk of Weight Loss After Esophageal Cancer Surgery

**DOI:** 10.1245/s10434-019-07352-5

**Published:** 2019-04-19

**Authors:** Anna Schandl, Joonas H. Kauppila, Poorna Anandavadivelan, Asif Johar, Pernilla Lagergren

**Affiliations:** 1 Surgical Care Science, Department of Molecular medicine and Surgery, Karolinska Institutet, Karolinska University Hospital, Stockholm, Sweden; 2 Cancer and Translational Medicine Research Unit, Medical Research Center Oulu, University of Oulu, Oulu University Hospital, Oulu, Finland; 3 Upper Gastrointestinal Surgery, Department of Molecular Medicine and Surgery, Karolinska Institutet, Karolinska University Hospital, Stockholm, Sweden; 4 Department of Surgery and Cancer, Imperial College, London, UK

## Abstract

**Background:**

Malnutrition after esophageal cancer surgery is associated with reduced health-related qualify of life. Therefore, a prediction model identifying patients at risk for severe weight loss after surgery was developed.

**Methods:**

Data from a Swedish population-based cohort study, including 616 patients undergoing esophageal cancer surgery in 2001–2005, was used. Candidate predictors included risk factors available before and immediately after surgery. Severe weight loss was defined as ≥ 15% loss of body weight between the time of surgery and 6 months postoperatively. The prediction model was developed using multivariable models. The accuracy of the model was measured by the area under the receiver operating characteristics curve (AUC) with bootstrap validation. The model was externally validated in a hospital-based cohort of 91 surgically treated esophageal cancer patients in the United Kingdom in 2011–2016. Each predictor in the final model was assigned a corresponding risk score. The sum of risk scores was equivalent to an estimated probability for severe weight loss.

**Results:**

Among the 351 patients with 6 months follow-up data, 125 (36%) suffered from severe postoperative weight loss. The final prediction model included body mass index at diagnosis, preoperative weight loss, and neoadjuvant therapy. The AUC for the model was 0.78 (95% CI 0.74–0.83). In the validation cohort, the AUC was 0.76. A clinical risk assessment guide was derived from the prediction model.

**Conclusions:**

This prediction model can preoperatively identify individuals with high risk of severe weight loss after esophageal cancer surgery. Intensive nutritional interventions for these patients are recommended.

Nutritional problems are common in esophageal cancer patients, with a significant proportion of patients already suffering from severe weight loss at diagnosis.^1^–^3^ Malnutrition after extensive gastrointestinal surgery has been associated with poor postoperative recovery, reduced survival rate, and deterioration in health-related quality of life (HRQOL).^4^–^7^ The underlying cause of such malnutrition is complex and multifactorial. The primary symptom of esophageal cancer is progressive dysphagia, caused by an obstructing tumor.^3^,^8^ This may lead to the increased basal metabolism not being met due to insufficient caloric intake, which results in weight loss. The weight loss also can be related to the cancer treatment. Chemoradiotherapy, which often is included in the esophageal cancer treatment, can contribute to catabolic muscle loss.^9^,^10^ During esophagostomy, a large part of the stomach is reconstructed into a tube, which replaces the removed esophagus and upper stomach.^11^ This may lead to the normal capacity of the stomach being lost. The altered anatomy with the missing reservoir function is usually accompanied by physiological issues with pancreatic insufficiency and/or gastric dumping syndrome and often presents as eating difficulties.^12^ There also is a psychological aspect of the problem, where the diagnosis and treatment can result in depressive symptoms, which may in turn further reduce appetite.^5^ To prevent or reduce such involuntary weight loss, a method for identifying patients at risk at an early postoperative stage would be beneficial for tailored follow-up planning. The at-risk patients could then be further screened to plan individually for nutritional support in those with poor nutritional status.^13^,^14^ The purpose of this study was to develop and externally validate a prediction model identifying patients at risk for severe weight loss after esophageal cancer surgery, by using objective parameters measured preoperatively or immediately after surgery.

## Methods

### Study Design

This is a nationwide and population-based cohort study, including all patients with curatively intended surgery for esophageal cancer in Sweden during a 5-year period, with follow-up for weight loss 6 months postoperatively, referred to later as the “development cohort.” A single-center, prospective cohort from St Thomas’ Hospital in London, UK, was used for validation, referred to later as the “validation cohort.” Informed consent was obtained from all participants and the Ethical Review Board at Karolinska University Hospital, Karolinska Institutet, Sweden, and the National Research Ethics Services in London and West Midlands (11/LO/0335 and 13/WM/0131) approved the study.

### Data Collection

Data in the development cohort were collected from the “Swedish Esophageal and Cardia Cancer” cohort study, where > 90% of Swedish patients undergoing curatively intended esophageal cancer surgery from 2001 until 2005 were included. More detailed information about the study design can be found in previous publications.^15^,^16^ The validation cohort was prospectively collected from the St Thomas’ Hospital in London, UK, including patients undergoing upper gastrointestinal cancer surgery between November 2011 and February 2016. More detailed information on the cohort can be found in a previous publication.^17^ In brief, the cohorts contained prospectively collected information regarding patient characteristics, tumor stage, type and location, surgical procedure, and complications. They also included self-reported information on weight at the time of surgery and 6 months postoperatively.

### Candidate Predictors

Variables considered for the prediction model were specified a priori and were identified from the literature and clinician input.^18^ The variables included patients’ age, female sex, comorbidities, body mass index, preoperative weight loss, tumor histology (adenocarcinoma), tumor location (lower esophagus or gastroesophageal junction), advanced tumor stage (III-IV), and neoadjuvant therapy.^1^,^19^–^21^

### Outcome Assessment

Postoperative weight loss was calculated as weight in kilograms (kg) at 6 months after surgery − weight in (kg) at the time of surgery. Patients were dichotomized into those with severe weight loss (≥ 15% weight loss) or not (< 15% weight loss). This cutoff was based on previous research showing that 10–15% of unintentional weight loss is predictive of poorer clinical outcomes for patients with cancer and thus reduces the possibility of successful postoperative recovery.^4^,^22^,^23^

### Statistical Analysis

Patient characteristics were displayed as numbers and percentages. The development of the prediction model for severe weight loss was done by examining the candidate predictors for univariate associations using a logistic regression model, including one covariate at a time. Variables with a *p* value exceeding 0.1 in the univariate analysis were excluded from further analyses, whereas the remaining variables were included in the multivariable model. The area under the receiver operating characteristics curve (AUC) was utilized as a measure of overall accuracy of the prediction model. Again, the candidate predictors were removed one at a time, and the AUC was recalculated each time until the most “optimal” model was reached.^24^ To further evaluate the predictive accuracy of the model, the AUCs were cross-validated in 1000 bootstrap samples. Results were presented as odds ratios (ORs) and 95% confidence intervals (CIs). The point estimate for each predictor included in the final model was equivalent to its associated risk for the severe postoperative weight loss. These point estimates were multiplied with respective predictors and then added to create a total score named “risk scores.” The external validation of the prediction model was done by applying the risk model on the validation cohort and calculating the AUC. All statistical analyses were conducted by a senior biostatistician (AJ) using SAS 9.4 (SAS institute Inc., Cary, NC).

## Results

### Patients

In the development cohort, 616 patients underwent esophageal cancer surgery of which 512 patients survived until the 6-month follow-up. Of these individuals, 383 (75%) reported their weight both at the time of the operation and 6 months postoperatively. Thirty-two patients were excluded because of incomplete data on candidate predictors. Among the 351 included patients, 125 (36%) suffered from ≥ 15% postoperative weight loss within 6 months of surgery (Table 1). Most patients were men with preoperative overweight. The dominant type of cancer was adenocarcinoma, located in the lower esophagus or gastroesophageal junction. Most patients received no neoadjuvant therapy and underwent transthoracic surgery.

**Table 1:** Characteristics of the 351 included patients in the development cohort, 6 months after esophageal cancer surgery

Characteristics	Categorization	Postoperative weight loss ≥ 15%	Postoperative weight loss < 15%
		Number (%)	Number (%)
Total		125 (100)	226 (100)
Age at operation (yr)	< 70	84 (67)	129 (57)
	≥ 70	41 (33)	97 (43)
Sex	Men	101 (81)	184 (81)
	Women	24 (19)	42 (19)
Comorbidity	0	73 (58)	127 (56)
	≥ 1	52 (42)	99 (44)
Body mass index (kg/m^2^)	< 25	37 (30)	147 (65)
	≥ 25	88 (70)	79 (35)
Preoperative weight loss (%)	< 10	116 (93)	150 (66)
	≥ 10	9 (7)	76 (34)
Tumor histology	Squamous cell carcinoma	21 (17)	59 (26)
	Adenocarcinoma and dysplasia	104 (83)	167 (74)
Tumor location	Lower esophagus or cardia	112 (90)	186 (82)
	Upper or middle esophagus	13 (10)	40 (18)
Tumor stage^a^	0–I	31 (25)	45 (20)
	II	39 (32)	71 (32)
	III	46 (37)	90 (40)
	IV	7 (6)	18 (8)
Neoadjuvant therapy	Yes	12 (10)	9 (4)
	No	113 (90)	217 (96)
Surgery type	Transthoracic	99 (79)	195 (86)
	Transhiatal	26 (21)	31 (14)

^a^Information about tumor stage was missing in four patients

In the validation cohort, a total of 91 patients underwent open transthoracic or transhiatal surgery for esophageal cancer survived at least six months after surgery and had complete information on the predictor and outcome variables. Thirty-five patients (38%) had ≥ 15% postoperative weight loss after surgery (Table 2). The dominant procedure in the validation cohort was transhiatal surgery (65%), and the majority underwent neoadjuvant chemotherapy (87%).

**Table 2:** Characteristics of the 91 patients included in the validation cohort, 6 months after esophageal cancer surgery

Characteristics	Categorization	Postoperative weight loss ≥ 15%	Postoperative weight loss < 15%
		Number (%)	Number (%)
Total		35 (100)	56 (100)
Age at operation (yr)	< 70	28 (80)	35 (63)
	≥ 70	7 (20)	21 (38)
Sex	Men	24 (69)	45 (80)
	Women	11 (31)	11 (20)
ASA-class	I–II	21 (60)	41 (73)
	III–IV	13 (37)	9 (16)
	Missing	1 (3)	6 (11)
Body mass index (kg/m^2^)	< 25	4 (11)	25 (45)
	≥ 25 kg/m^2^	31 (89)	31 (55)
Preoperative weight loss (%)	< 10	29 (83)	36 (64)
	≥ 10	6 (17)	20 (36)
Tumor histology	Squamous cell carcinoma	5 (14)	4 (7)
	Adenocarcinoma and dysplasia	30 (86)	52 (93)
Tumor location	Lower esophagus or cardia	35 (100)	54 (96)
	Upper or middle esophagus	0 (0)	2 (4)
Tumor stage^a^	0-I	4 (11)	8 (14)
	II	11 (31)	11 (20)
	III	20 (57)	37 (66)
Neoadjuvant therapy	Yes	33 (94)	46 (82)
	No	2 (6)	10 (18)
Surgery type	Transthoracic	11 (31)	21 (38)
	Transhiatal	24 (69)	35 (63)

^a^American Society of Anesthesiologists

### Development of the Prediction Model

Of the original nine candidate predictors, six [age, body mass index (BMI), preoperative weight loss, tumor histology, tumor location, and neoadjuvant therapy] were found to be sufficiently associated with ≥ 15% weight loss 6 months after surgery (*p* < 0.1) in the univariate logistic regression analyses to merit inclusion in the multivariable regression model (Table 3). Thus, sex, comorbidity, and tumor stage were excluded from further analysis. In the following analysis, age, tumor histology, and tumor location were removed from the model, because these candidate predictors increased the AUC by < 1% point, which was not considered clinically relevant. The final multivariable prediction model consisted of three predictors associated with ≥ 15% weight loss 6 months after surgery: BMI [odds ratio (OR) 1.13; 95% confidence interval (CI) 1.05–1.21], preoperative weight loss (OR 0.92; 95% CI 0.89–90.96), and neoadjuvant therapy (OR 2.02, 95% CI 0.71–5.74; Table 4). The point estimate of each predictor corresponded to an estimated probability of ≥ 15% weight loss.

**Table 3:** Candidate predictors and their univariate associations with the risk of ≥ 15% weight loss 6 months after esophageal cancer surgery presented as regression coefficients, odd ratios (ORs) with 95% confidence intervals (CIs) and *p* values

Candidate predictors	Description (reference)	Regression coefficient	Crude OR (95% CI)	*p* value
Age	Continuous	− 0.03	0.97 (0.95–0.99)	0.006
Sex	Female	0.05	1.06 (0.63–1.78)	0.838
Comorbidity	≥ 1	− 0.26	0.77 (0.51–1.18)	0.230
Body Mass Index	Continuous kg/m^2^	0.22	1.25 (1.18–1.33)	< 0.001
Pre-operative weight loss	Continuous	− 0.11	0.89 (0.87–0.92)	< 0.001
Tumor histology	Adenocarcinoma and dysplasia	0.54	1.71 (1.03–2.84)	0.038
Tumor location	Lower esophagus and cardia	0.63	1.88 (1.02–3.45)	0.042
Tumor stage	III–IV	− 0.12	0.89 (0.58–1.34)	0.563
Neoadjuvant therapy	Yes	1.07	2.92 (1.23–6.92)	0.015

**Table 4:** Final multivariable prediction model for identifying patients with ≥ 15% weight loss 6 months after esophageal cancer surgery, presented as regression coefficients, odds ratios (ORs) with 95% confidence intervals (CIs) and estimated risk scores

Predictors	Description (reference)	Regression coefficient	OR (95% CI)
Body mass index	Continuous	0.12	1.13 (1.05–1.21)
Preoperative weight loss	Continuous	− 0.08	0.92 (0.89–0.96)
Neoadjuvant therapy	No	0.70	2.02 (0.71–5.74)

Logistic regression equation: log-odds of weight loss = −3.51 + (0.12*BMI) − (0.08*Preoperative weight loss) + (0.70* Neoadjuvant therapy)

### Validation of the Prediction Model

**Fig. 1:**
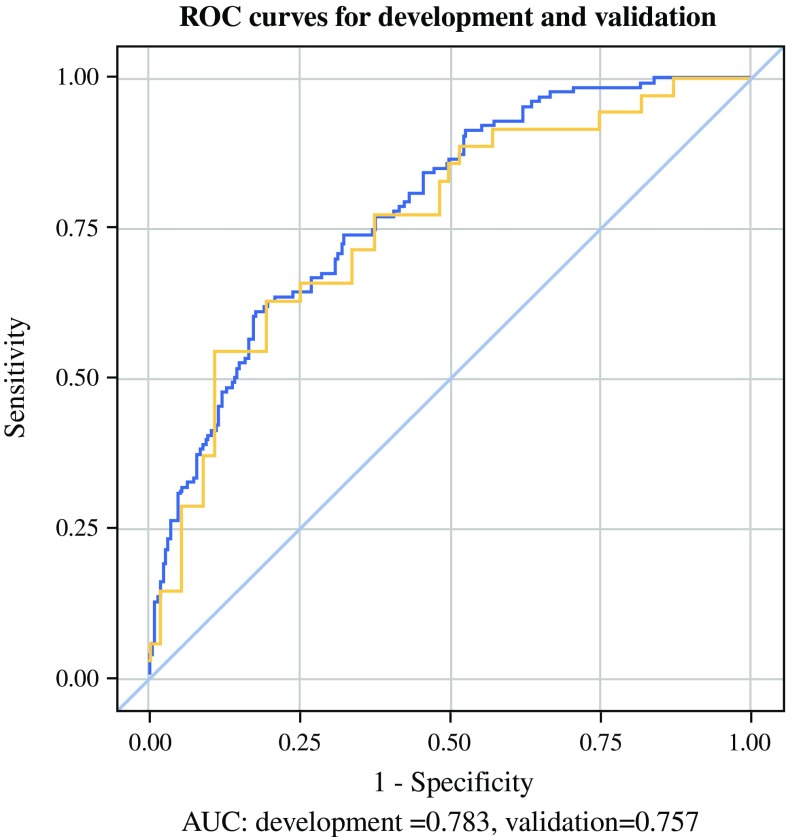
Receiver operating characteristics curves based on the final prediction model for > 15% weight loss within 6 months of esophageal cancer surgery in the development cohort and in the validation cohort

The AUC for the performance of the final prediction model in the development cohort was 0.78 (95% CI 0.74–0.83; Fig. 1). This result was confirmed in a bootstrap cross-validation of 1000 samples, where the AUC was found to be 0.786 (95% CI 0.785–0.787). In the validation cohort, the AUC for the performance of the prediction model formed in the development cohort was 0.76 (Fig. 1).

### Translation to Clinical Practice

To obtain a risk score feasible for use in clinical practice, each predictor was assigned a weight equal to the corresponding logistic regression model coefficient and predicted probability was calculated, which is used as a risk score. Based on the development cohort, 82% patients with risk score > 0.7, 62% patients with risk score between 0.6 and 0.7, 55% patients with risk score between 0.5 and 0.6, and 27% patients with risk score < 0.5 had weight loss ≥ 15%. In the external validation on the UK cohort, 57% patients with risk score of > 0.5 had weight loss ≥ 15% with sensitivity and specificity of 71% and 67%, respectively. Therefore, the risk score of > 0.5 would make a patient a “high-risk” patient for postoperative weight loss of ≥ 15% and would be recommended for intensified nutritional intervention.

## Discussion

In this study, a risk prediction model for postoperative weight loss after esophageal cancer surgery was developed and validated by including three predictive variables: high BMI at diagnosis, less preoperative weight loss, and neoadjuvant therapy.

A main strength of this study is the use of nationwide and population-based data for the development of the model, which reduces the risk of selection bias and increases the generalizability of the results. Another advantage is the availability of many clinically relevant variables for evaluation in the prediction model. The selection of clinical variables is crucial when developing prediction models for clinical use.^18^ Therefore, only variables that were in line with daily clinical practice were included. The selection was based on relevant literature, and only variables feasible for evaluation at a consultant visit were chosen. Inevitably, some potentially relevant variables may have been missed. The outcome of this study was based on self-reported data, which by its nature induces a risk for misclassification bias. However, such biases would be nondifferential and equally distributed between patients with severe weight loss and not. Moreover, the validity of the self-reported weight data for the development cohort has previously been investigated and found to be of high accuracy.^25^ The proportion of patients with severe weight loss is dependent on the chosen cutoff level for the outcome measure. Our choice was based on literature that defines severe nutritional risk as the presence of > 10–15% within 6 months, and we chose the higher limit of this range to make the prediction more robust. In terms of predictive accuracy, the model had an AUC of 0.78 in the bootstrap cross-validation, which compares favorably with other well-known cancer risk models.^26^ However, internal validation may result in an overoptimistic assessment of the model’s performance, and therefore the model was validated externally in an independent prospective cohort from another country with high proportions of patients undergoing transhiatal surgery and contemporary neoadjuvant chemotherapy, still showing a fair predictive performance with AUC of 0.76. Finally, although a total of 603 patients participated in the current study, a larger sample would likely have improved the accuracy of the model.

Obesity and overweight are main risk factors for developing esophageal adenocarcinoma.^27^,^28^ For some of these patients, weight reduction can be associated with positive outcomes. However, when the weight loss relates to muscles and not fat, it may have a negative impact on patients’ physical function. Because many esophageal cancer patients report deterioration in physical function, it can be assumed that at least part of the weight loss may result from muscle loss.^29^ Currently, in most hospitals, there is no routine procedure for meeting a dietician after esophageal cancer surgery despite international recommendations. The surgeon usually informs patients of the overall risk of weight loss, but depending on the surgeon’s knowledge and hospital site, the content of information or suggested interventions may differ. Some patients with low risk for weight loss may likely recover uneventfully, whereas others with a higher risk may suffer from severe problems. For the clinician, a risk prediction model can help to identify these susceptible patients and support decisions about treatment strategies. Depending on hospital resources, the choice of which risk probability level to use for interventions may vary. By excluding patients with low risk for postoperative weight loss, a smaller number of patients remain for more resource-intensive assessment and therapy. High-risk patients would most likely benefit from early assessment of nutritional problems, such as taste and smell alterations, constipation, mucositis, pain, dyspnea, as well as intensive interventions, such as dietary advice, oral supplementation, or enteral feeding with tube replacement in combination with exercise training.^14^,^30^,^31^ Such interventions were designed to improve weight gain, performance status, tolerability of treatment, quality of life, and overall survival in cancer patients. Patients falsely identified as at risk will not be exposed to any hazardous interventions, and therefore the developed risk prediction model can be readily used in clinical practice.

In summary, important predictors for weight loss in esophageal cancer surgery patients were used to develop and validate a prediction model for identifying patients susceptible to severe weight loss. The final model includes the variables high BMI, less preoperative weight loss, and neoadjuvant therapy. The prediction model can be readily used to triage high-risk individuals to intensified nutritional and exercise programs and to develop other strategies mitigating weight loss after esophagectomy. Further testing of the validity of the model in external populations and estimating the impact of various intervention strategies for weight loss is encouraged.
